# Characteristics of Hypotonic–Hyporesponsive Episodes (HHEs) Following Childhood Vaccination: A 13-Year Analysis of Spontaneous Reports to the Dutch Pharmacovigilance Centre Lareb

**DOI:** 10.3390/vaccines14060547

**Published:** 2026-06-20

**Authors:** Sanne Boetzkes, Leontine van Balveren, Florence van Hunsel

**Affiliations:** 1 Pharmacovigilance Centre Lareb, Goudsbloemvallei 7, 5237 MH ‘s-Hertogenbosch, The Netherlands; s.boetzkes@lareb.nl (S.B.); l.vanbalveren@lareb.nl (L.v.B.); 2 Department of Pharmacotherapy, -Epidemiology & -Economics, Groningen Research Institute of Pharmacy (GRIP), University of Groningen, Antonius Deusinglaan 1, 9713 AV Groningen, The Netherlands

**Keywords:** HHE, hypotonic hyporesponsive episode, national immunisation programme (NIP), hexavalent DTaP IPV Hib-HB vaccine, pneumococcal vaccine, adverse event following immunisation (AEFI)

## Abstract

**Background:** Hypotonic–hyporesponsive episode (HHE) is a recognised adverse event following immunisation (AEFI) in infants, characterised by sudden hypotonia, hyporesponsiveness, and pallor or cyanosis. Although considered benign, its abrupt and often dramatic presentation often leads to acute medical evaluation. Contemporary data on HHE are limited, and awareness among healthcare professionals needs attention. **Methods:** We conducted a retrospective analysis of all spontaneous reports of HHE submitted to the national pharmacovigilance centre Lareb between 1 January 2012 and 22 July 2025. Cases were included only when meeting Brighton Collaboration (BC) Level 1 criteria, requiring clear documentation of hypotonia, hyporesponsiveness, and pallor or cyanosis in children younger than 24 months. Demographic and clinical characteristics, vaccine combinations, latency, duration, seriousness, and medical care utilisation were described. **Results:** A total of 294 Level 1 HHE cases were identified. Most episodes followed combinations of hexavalent vaccines with pneumococcal conjugate vaccines. The median age at onset was 9 weeks, with slightly more reports involving boys. The median latency to onset was 5 h (range 4–8 h), and the median episode duration was 10 min (range 3–30 min), aligning with the historical literature. All children recovered fully, and no long-term sequelae were reported. Although HHE is clinically benign, 27% of cases were classified as serious, primarily due to hospital admission. Among non-serious cases, one third involved medical assessment or emergency services. Healthcare professionals submitted 44% of reports, notably community child health physicians. **Conclusions:** Contemporary Dutch pharmacovigilance data confirm that the clinical characteristics of HHE remain highly consistent with long-standig evidence. Despite its benign and self-limiting nature, HHE frequently triggers substantial medical care consumption. Improved awareness of the typical presentation, course, and prognosis, supported by the Brighton Collaboration criteria, may help clinicians recognise HHE more readily, reduce unnecessary medical consumption, and provide reassurance to caregivers.

## 1. Introduction

Hypotonic–hyporesponsive episode (HHE) is an adverse event following immunisation (AEFI) that affects infants and young children under two years of age. It is characterised by the sudden onset of hypotonia, hyporesponsiveness, and pallor or cyanosis, typically occurring within hours after vaccination and resolving spontaneously without residual effects [1]. Although HHE is considered a benign and self-limiting clinical entity, its presentation can be alarming for parents and healthcare professionals. As a result, HHE frequently prompts medical evaluation, and in some cases, emergency care or hospital admission.

The majority of the scientific literature describing HHE dates from the 1990s to the early 2000s [2,3,4,5,6,7,8,9,10]. Most widely cited estimates of latency and episode duration originate from an analysis of reports submitted to the Vaccine Adverse Event Reporting System (VAERS) in 2000 [9]. Since then, only limited contemporary data on latency and episode duration have been published. The last comprehensive case definition, developed by the Brighton Collaboration (BC), was released in 2007 [6]. This definition remains the international standard for surveillance and research. According to a postmarketing safety study performed by Hansen et al. [11] on data from 2002 to 2005, the estimated overall risk of HHE after either of the first two doses of DTaP vaccination within the National Immunisation Programme (NIP) at the age of 2 and 4 months was 44 per 100,000. A few studies investigated the recurrence risk of HHE which was generally low and ranged from 0 to 3% [2,12,13].

In The Netherlands, spontaneous reports of a suspected adverse event following immunisation (AEFIs) are collected by the national pharmacovigilance centre Lareb. Recurrent inquiries to the Lareb helpdesk and observations from spontaneous reports suggest that HHE may not always be recognised or correctly classified by healthcare professionals. In some cases, reporters describe only individual symptoms or apply alternative diagnostic labels, such as Brief Resolved Unexplained Event (BRUE). This under-recognition may contribute to uncertainty among clinicians and caregivers when encountering HHEs, resulting in unnecessary diagnostic procedures or referrals.

We conducted a comprehensive analysis of all spontaneous reports of HHE submitted to Lareb between 2012 and 2025. Using the Brighton Collaboration criteria, we aimed to describe the clinical characteristics of HHE, compare observed latency and duration with previously published data, and provide clinically relevant information to support recognition and management of HHE by healthcare professionals. Given the benign nature of HHE and the high level of concern in both parents and clinicians, updated data may help improve awareness and reduce misclassification.

## 2. Materials and Methods

### 2.1. Safety Surveillance System

The Dutch National Immunisation Programme (NIP) is organised by the National Institute for Public Health and Environment (RIVM). The NIP has been modified at various points in the past, largely driven by the introduction of updated vaccine formulations and adjustments in vaccine combinations [14,15].

Since 2012, the safety of vaccines used in the NIP is monitored continuously by passive surveillance through the spontaneous reporting system maintained by the national pharmacovigilance centre Lareb [16]. The reports consist of symptoms that occurred after vaccination, for which the reporter suspects a possible adverse reaction. These reported reactions therefore fall under the international definition of an adverse event following immunisation (AEFI): an undesirable event occurring after passive or active vaccination, which does not necessarily have a causal relationship with the administered vaccine(s) [17]. The spontaneous reporting system allows both vaccinated individuals (or their parents/caregivers) and healthcare professionals to report a suspected adverse reaction. The reporting form collects personal data, vaccination details, the course of the symptoms, and other potential contributing factors. Additional information is requested from the reporter when necessary. All AEFIs are coded according to the Medical Dictionary for Regulatory Activities (MedDRA^®^) [18], using the most specific clinical codes possible based on the reported information.

### 2.2. HHE Case Definition

Hypotonic–hyporesponsive episodes (HHEs) were classified according to the BC case definition, which provides standardised criteria for the diagnosis and reporting of HHE in infants and young children [6]. The BC definition restricts the diagnosis to children under 24 months of age, as older children with a similar presentation are more likely to experience vasovagal syncope, which differs in pathophysiology and time to onset [6,10]. The BC definition is based on the presence of a characteristic clinical triad and assigns cases to one of three levels of diagnostic certainty.

An HHE is characterised by the sudden onset of the following three clinical signs:

1. Hypotonia—a marked decrease in muscle tone, causing the infant to become limp or “floppy”.
2. Hyporesponsiveness—reduced or absent responsiveness to external stimuli, often described as diminished consciousness.
3. Pallor or cyanosis—acute pallor or bluish discoloration of the skin, typically around the face or perioral region.

These manifestations are typically transient and resolve spontaneously without sequelae.

The BC case definition distinguishes three levels of diagnostic certainty to accommodate differences in clinical documentation.

Level 1 (highest certainty) requires clear documentation of all three components of the triad: hypotonia, hypo- or unresponsiveness, and pallor/cyanosis.

Level 2 (intermediate certainty) is assigned when one component of the triad is not documented, but the remaining two provide a clinical picture consistent with HHE.

Level 3 (lowest certainty) applies when documentation is more limited but the presentation remains suggestive of HHE.

For the present analysis, we included only cases that met Brighton Collaboration Level 1 criteria. This ensured a uniform and stringent case definition for all included spontaneous reports which is in line with other studies [3,5,7]. Brief Resolved Unexplained Event (BRUE) is included in the differential diagnosis of HHE. Although children with BRUE may also present with the triad of pallor, hypotonia, and decreased consciousness, this group is additionally characterised by disturbed respiration [19]. Moreover, HHE consistently occurs in association with vaccination, whereas BRUE is generally not linked to vaccination. We received a few reports from paediatricians in which the diagnosis of BRUE was initially recorded. However, upon further contact with these physicians and more detailed discussion of the cases, some were ultimately identified as HHE, after which the diagnosis was revised in consultation with the reporting clinician. These reports partly motivated us to conduct this study, with the aim of increasing awareness of the concept of HHE.

### 2.3. Study Design and Inclusion Criteria

A retrospective descriptive analysis was conducted on all reported cases coded as hypotonic–hyporesponsive episode (MedDRA PT) or meeting Brighton Collaboration (BC) Level 1 criteria received between 1 January 2012 and 22 July 2025. All reports containing the reaction HHE, or the reactions hypotonia, pallor, and/or hyporesponsiveness to stimuli following administration of a vaccine within the National Immunisation Programme in children under 2 years of age, were collected. As all reports had previously been reviewed by a scientific assessor, a second review of the selected reports by a single scientific assessor (an MD with a background in paediatric medicine) was deemed sufficient. In total, three reports coded as HHE lacked sufficient information to meet Level 1 criteria according to the Brighton Collaboration. These reports were therefore excluded from the analysis after discussion with a second assessor.

Collected variables include administered vaccine(s), age and sex of the children, relevant medical history, concomitant medication, latency period, duration of episode, healthcare resource utilisation and reporter type (healthcare professional vs. consumer). For each case report it was assessed whether it met the criteria for a serious event according to the definition of the Council for International Organizations of Medical Sciences (CIOMS) [20].

### 2.4. Data Analysis

Descriptive statistics were used to summarise demographic and clinical characteristics. Medians, means, ranges, and percentages were reported when appropriate. A ridgeline plot (R Core Team (2026), version 4.6. 0) was used for visualisation of latency time and duration. No incidence or frequency estimates were rated due to limitations in spontaneous reporting systems.

## 3. Results

### 3.1. Vaccine Combinations

Most episodes occurred after vaccine combinations containing a hexavalent DTaP-IPV-Hib-HB vaccine and pneumococcal vaccine, reflecting the standard immunisation schedule in The Netherlands during the study period. Table 1 shows vaccine combinations associated with HHE reports (*n* = 294).

Generic vaccine types correspond to their vaccine product name types as follows: hexavalent DTaP-IPV-Hib-HB vaccines (Infanrix hexa^®^: GlaxoSmithKline (GSK), Rixensart, Belgium; Vaxelis^®^: Merck Sharp & Dohme B.V. (MSD), Leiden, The Netherlands); pneumococcal conjugate vaccines (Synflorix^®^: GlaxoSmithKline (GSK), Rixensart, Belgium; Vaxneuvance^®^: Merck Sharp & Dohme B.V. (MSD), Haarlem, The Netherlands); rotavirus vaccine (Rotarix^®^: GlaxoSmithKline (GSK), Rixensart, Belgium); measles–mumps–rubella vaccine (MMRvaxPro^®^: Merck Sharp & Dohme B.V. (MSD), Haarlem, The Netherlands); and meningococcal vaccines (Neisvac-C^®^: Pfizer B.V., Capelle aan den Ijssel, The Netherlands; Nimenrix^®^: Pfizer Europe MA EEIG, Brussel, Belgium).

### 3.2. Case Characteristics

A total of 294 reports met the inclusion criteria. Boys were slightly overrepresented; 58% were male (*n* = 170) and 42% female (*n* = 124).

The median age at onset was 9 weeks (range 5–68 weeks), with the majority of children (71%) presenting between 8 and 15 weeks of age.

Relevant medical history was infrequently reported and included prematurity (*n* = 4), cow’s milk protein allergy (*n* = 3), a previous HHE (*n* = 2), congenital hypothyroidism (*n* = 1) and laryngomalacia (*n* = 1).

The same applies to the reported use of concomitant medication, which was limited to acetaminophen (*n* = 4), ferrous fumarate (*n* = 1), ferrous chloride (*n* = 1), lactulose (*n* = 1), levothyroxine (*n* = 1) and salbutamol (*n* = 1).

### 3.3. Latency and Duration

Latency was known for 292 reports, with a median latency of 5 h (range 4–8 h). However, the overall range extended from 1 h to 216 h. Even though HHE is expected to occur hours after vaccination according to the current literature [9], in this study, we examine AEFIs for which we cannot a priori exclude that reactions may occur later than expected. Taking into account the IQR (interquartile range), the outliers in latency are less than 2 h (38 in total) and more than 10 h (44 in total). If we exclude these outliers the median latency of the remaining 209 reports is also 5 h with an average of 5.3 h.

**Figure 1:**
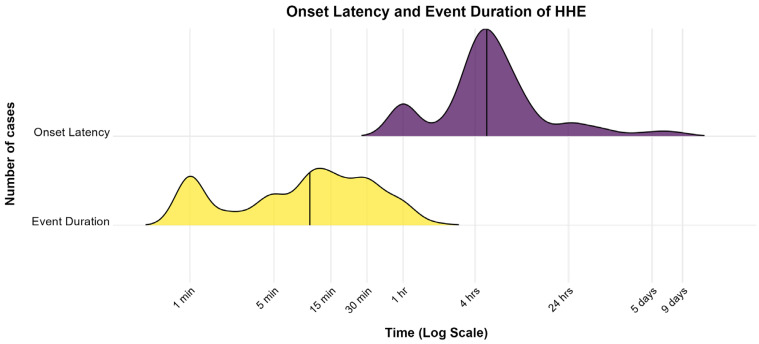
Latency of HHE in hours (*n* = 292). The median latency was 5 h with a range of 0–216 h. Most latencies were between 4 and 8 h. Duration of HHE (*n* = 215) in minutes. The median duration was 10 min with a range of 1–120 min. Most durations were between 3 and 30 min.

Duration was available for 215 reports, with a median duration of 10 min (range 3–30 min) (Figure 1).

### 3.4. Seriousness and Healthcare Resource Consumption

A total of 79 reports (27%) met CIOMS criteria for seriousness, predominantly due to hospital admission. The definition of hospital admission in CIOMS is a minimum stay of 24 h. In the majority of these serious reports due to hospital admission, children were admitted to the hospital for 1 to 2 days for observation. Since 2012, the national pharmacovigilance centre Lareb has published the total amount of reports and serious reports following administration of a vaccine within the National Immunisation Programme, per year [21]. During the study period a total amount of approximately 20,000 reports were received of which 1076 (5.5%) were serious according to the CIOMS criteria. This means 7.3% of all serious reports contained the AEFI HHE. Among the non-serious cases, 72 (33%) still involved evaluation by a healthcare provider, including general practitioners (*n* = 37), ambulance dispatch (*n* = 19), emergency department or paediatric evaluation (*n* = 10) and community child health physician assessment (*n* = 6).

### 3.5. Reporter Type

Reporting patterns differed from typical vaccine-related AEFI submissions. Consumers or parents submitted 56% of reports. Healthcare professionals accounted for the remaining 44% of reports, with community child health physicians representing 57% of these. These numbers are substantially higher than expected for routine childhood vaccine reporting in The Netherlands, where healthcare professionals were responsible for an average of 21% of the reports in the period between 2012 and 2025 [21].

## 4. Discussion

In this nationwide analysis of spontaneous reports submitted to the national pharmacovigilance centre Lareb, we characterised 294 cases of hypotonic–hyporesponsive episode (HHE) following routine childhood vaccination over a 13-year period. To our knowledge, this represents one of the most detailed and contemporary analyses of HHE since the early 2000s. The clinical profile observed in these reports, particularly regarding latency and episode duration, confirms the definition of HHE as an acute, self-limiting, and benign reaction that occurs primarily in infancy.

The median latency of approximately 5 h and typical range of 4–8 h closely mirror the most frequently cited data from earlier studies. Similarly, the median episode duration of 10 min (range 3–30 min) aligns with historical observations, including the analysis by DuVernoy and Braun, although our dataset suggests that episodes may tend to be somewhat shorter than previously reported [3,4,9]. Despite the age of the earlier literature, these findings underscore that the classical clinical characteristics of HHE have remained remarkably consistent over time.

Importantly, all children in our dataset experienced full spontaneous recovery, and no cases of residual deficits or long-term adverse outcomes were reported. This supports the prevailing view that HHE is a benign phenomenon, even though its sudden and serious presentation can cause significant concern among caregivers and healthcare professionals.

A notable finding of this study is the relatively high proportion of cases classified as serious according to CIOMS criteria (27%). Seriousness was most often assigned due to hospital admission, rather than clinical severity. The average age in both the serious group and the non-serious group was around 16 weeks, so seriousness does not seem to be determined by age. Among the non-serious reports, a substantial proportion still involved medical evaluation, including general practitioner assessment, emergency department visits, and ambulance dispatch. These patterns likely reflect the alarming nature of the episode, rather than underlying clinical risk. The high level of medical care consumption emphasises the need for better awareness of HHE among clinicians, especially those providing acute paediatric care, to support timely recognition and avoid unnecessary investigations.

The distribution of reporter types in our dataset is remarkable, but somehow expected. Whereas most spontaneous vaccine-related adverse event reports in The Netherlands are submitted by consumers, HHE reports were nearly equally submitted by healthcare professionals and consumers. Community child health physicians accounted for more than half of the professional reports, suggesting that physicians working in routine vaccination programmes are frequently involved in the initial evaluation of these events. This may reflect a greater tendency among professionals to report episodes that appear severe or alarming, or a greater familiarity with reporting pathways. Conversely, some cases may be misclassified as other events, particularly BRUE, when the clinical triad of HHE is not widely recognised.

The pathophysiology of HHE remains incompletely understood. Earlier studies have not identified consistent associations with endotoxin load, specific vaccine components, metabolic abnormalities, or electrophysiological changes [4,5]. The most commonly proposed mechanism is a transient vasomotor/autonomic reaction following immunisation [3,4,5]. The study of Vermeer-de Bondt [5] illustrates the impact of inflammation on the autonomic nervous system. There is no evidence that HHE is the direct result of autonomic nervous dysfunction. However it is suggested that in some infants, inflammation—as a result of vaccination—could provoke an increase in autonomous nervous system reactivity, which may result in vasomotor effects leading to the symptoms observed in HHE. Vermeer and-de Bondt [5] stated that advancing the vaccination schedule to an earlier age led to an increase in numbers of HHE. In the NIP the vaccination schedule was only recently revised (in 2020) from administration of the first vaccinations at 2 months of age to administration at 3 months of age. The majority of our data originates from the period in which vaccinations were administered at the age of 2 months. The median age of onset was 9 weeks with most children presenting between 9 and 15 weeks of age. This suggests that HHE predominantly occurs at a younger age or following administration of the initial vaccinations. A hypothesis is that an imbalance in the autonomic nervous system occurs more frequently in the youngest group of infants as this group has a less developed system. Possibly the threshold for hyperreactivity is lower compared to older infants.

This study has several strengths, including the relatively large sample size, the long observation period, and the strict application of Brighton Collaboration Level 1 criteria, ensuring high diagnostic specificity. However, limitations inherent to spontaneous reporting systems must be considered. We could not estimate the incidence or risk of HHE or use any other descriptive statistics because the spontaneous reporting system does not take into account how many vaccines have been administered within the NIP for example [22]. However, taking into account that vaccination coverage for children up to the age of two years in The Netherlands has diminished from approximately 90% in 2012 to 85% in 2025, that most children received approximately 10 doses of vaccines in the first two years and that a Dutch birth cohort consists of 170,000 children per year, in the period 2012–2025 the estimated amount of administered vaccines was roughly 19 million [23]. The fact that only 294 cases of HHE were reported underlines the rareness of the phenomenon, even when we take underreporting, which is well known in spontaneous reporting in pharmacovigilance, and temporal changes in reporting practices over the years into account [24].

Missing information for some variables (particularly duration) may also have influenced the precision of our findings. Our data includes a small number of reports with a latency of more than 24 h which is quite long for an HHE to occur after vaccination. These reports may reflect a form of misclassification. Also, duration is missing in 79 of 294 reports. Unfortunately, missing data are inherent to a spontaneous reporting system, despite our best efforts to ensure that the data are as complete as possible [22]. Since the surveillance system is based on spontaneous reports, not all case reports are complete. Not all parameters are mandatory to complete in the reporting form. Where possible, follow-up information is requested from the reporter in cases of incomplete data; however, reporters are not obligated to respond, and in some instances, the information is simply not available to them. The duration of symptoms is an example of a parameter that is frequently missing in spontaneous reports [22]. The retrospective character of the study implies we have to work with the available information in our database. In line with these findings, we also see clustering of latency around 1 h and clustering of duration around 1 min after the event in Figure 1. This can be explained by the inaccuracy with which individuals recall and report data while reporting AEFIs [25].

Finally, the increased recognition and reporting of HHE by Dutch community child health physicians may not be generalizable to other countries.

Despite these limitations, our findings provide valuable contemporary data that may support improved recognition and management of HHE in clinical practice. Considering the benign nature of HHE and the high level of concern it can provoke, increased awareness among parents, community child health professionals, general practitioners, paediatricians and emergency care providers is essential. Clear guidance on the typical presentation, expected course, and recommended response may reduce unnecessary diagnostic interventions and alleviate anxiety.

## 5. Conclusions

In this contemporary analysis of spontaneous reports to the Dutch pharmacovigilance centre, the clinical characteristics of hypotonic–hyporesponsive episodes (HHEs) following routine childhood vaccination were highly consistent with long-standing descriptions in the literature. The observed latency of onset and episode duration closely matched previously published data, reinforcing the understanding of HHE as a transient, self-limiting, and benign event that occurs predominantly in young infants and resolves without sequelae. Although the clinical course is mild, the presentation is often alarming to caregivers and healthcare professionals, which likely explains the substantial proportion of cases classified as serious due to hospital admission and the high overall use of medical services.

Our findings underscore the importance of recognition and correct classification of HHE, particularly in settings where the diagnosis may be unfamiliar or confused with other acute events such as BRUE or vasovagal syncope. Improved awareness among clinicians, especially those working in primary care, paediatrics, and emergency medicine, may help reduce unnecessary concern, diagnostic testing, and healthcare utilisation. Pharmacovigilance centres, including Lareb, may play a key role in disseminating clear, evidence-based information to both healthcare professionals and parents.

## Figures and Tables

**Table 1:** Vaccine combinations associated with HHE reports (*n* = 294).

Vaccine (Combination)	*n*	% of Total
hexavalent DTaP-IPV-Hib-HB vaccine + pneumococcal conjugate vaccine	227	77%
hexavalent DTaP-IPV-Hib-HB vaccine	37	13%
hexavalent DTaP-IPV-Hib-HB vaccine + pneumococcal conjugate vaccine + rotavirus vaccine	16	5%
measles–mumps–rubella vaccine + meningococcal vaccine	8	3%
hexavalent DTaP-IPV-Hib-HB vaccine + rotavirus vaccine	3	1%
rotavirus vaccine	2	1%
measles–mumps–rubella vaccine	1	<1%

## Data Availability

The datasets generated and analysed during the current study are not publicly available due to the General Data Protection Regulation (GDPR) and the general privacy regulation of the pharmacovigilance centre Lareb, but are available from the corresponding author on reasonable request. The underlying code for this study is not publicly available but may be made available to qualified researchers on reasonable request from the corresponding author.

## Abbreviations

The following abbreviations are used in this manuscript:

AEFI	Adverse Event Following Immunisation
BC	Brighton Collaboration
HHE	Hypotonic–hyporesponsive episode
